# Patient reported outcomes for preschool children with recurrent wheeze

**DOI:** 10.1038/s41533-019-0120-3

**Published:** 2019-03-26

**Authors:** Makrinioti Heidi, Keating Emily, Holden Benjamin, Coren Michael, Klaber Robert, Blair Mitch, Griffiths Chris, Watson Mando, Bush Andrew

**Affiliations:** 1 Department of Paediatrics, St Mary’s Hospital, Imperial College Healthcare NHS Trust, London, UK; 2 Department of Paediatrics, Northwick Park Hospital, North West London Hospitals NHS Trust, London, UK; 3 Division of Medicine, Imperial College, London, UK; 4 Asthma UK Centre for Applied Research, Centre for Primary Care and Public Health, Blizard Institute, Barts and the London, School of Medicine and Dentistry, London, E1 2AT UK; 5 National Heart and Lung Institute, Imperial College, London, UK

## Abstract

Children with preschool wheeze regularly attend UK emergency departments. There is no international consensus on any specific personalised management approach. This paper describes the first attempt to co-design patient-centred outcomes with families. Preschool wheezers’ parents participated in semi-structured interviews and focus-group discussions to air their concerns and identify potential additional support. Fifty-seven families participated in these interviews. From these, themes were defined through qualitative content analysis. Parental experience was mapped to the patient pathway and seven important personalised outcomes were described. These can be used to inform a tool which following further validation could potentially support management of children with preschool wheeze and provide an additional patient focused clinical outcome measure in audit and research.

## Introduction

Chronic respiratory conditions are major causes of morbidity and mortality in childhood. Such children may have impaired physical, emotional, and general well-being. Their illness and associated stressors can have a great impact on their caregivers, especially when recurrent hospitalisations are required^1^.

Recurrent wheeze in preschool children is one of the most common respiratory conditions. Wheezing disorders in toddlers constitute one-third of the presentations of respiratory disorders in this age group and are associated with increased healthcare costs, loss of time from work in parents and impaired quality of life for the carers and/or the family^2,3^ The prevalence of preschool wheeze varies between countries. A recently published European study describes the differences in prevalence during the second year of life in nine European countries with UK having the second highest prevalence^4^.

In the UK, the last published national audit shows that the number of hospital admissions for preschool children with wheeze remained steadily high in the previous decade^5^ Recently, the first UK study looking at exacerbation rates in a general asthma population between the years 2007 and 2015 showed that the patients with the most frequent exacerbations were the group of children under 5 years old^6^ In Canada, the annual rate of emergency department visits is 23–42 per 1000 for preschool children with wheeze, compared with less than 15 per 1000 for those aged above six years old^7^ There is a similar pattern for the rate of hospital admissions.

A recent Australian study shows that nearly a third of children who present at the emergency department with a wheeze attack are discharged within 4 h and more than 40% are discharged within 7 h^8^, which has been reported in other studies^9,10^ The brevity of stay highlights that a significant number of preschool wheezers need not have sought clinical review at a secondary care setting. If we can understand why parents often seek hospital advice unnecessarily, more meaningful interventions for these patients can potentially be designed.

Patient-reported outcome measures (PROMs) are tools measuring outcomes that matter to patients. Many have been developed over the past 30 years but few are used routinely in clinical practice. Evidence shows that the systematic use of PROMs leads to better communication and decision making by doctors and patients and improves patient satisfaction and outcomes of care^11^ However, there are no UK PROMs for preschool children with wheeze, potentially impairing parental communication with doctors and coordination of their child’s care. We also contacted experts in management of preschool wheeze in eight different countries (UK, US, Argentina, Greece, Australia, Italy, Singapore, India) in order to identify whether any PROMs are used routinely in each country for the management of these children. We found that no PROMs have been routinely introduced in these countries either^12^ There are two instruments attempting to assess the severity of preschool wheeze attacks and the main parental concerns during the episode^13,14^ Although these instruments demonstrate how families feel during acute attacks, they do not capture the changes in quality of their life over time and are not co-designed with families as would a PROM tool^15^ Therefore, a PROM would potentially add significantly in the management of these children.

This paper describes the main co-developed personalised outcomes for preschool children with wheeze.

## Results

**Fig. 1:**
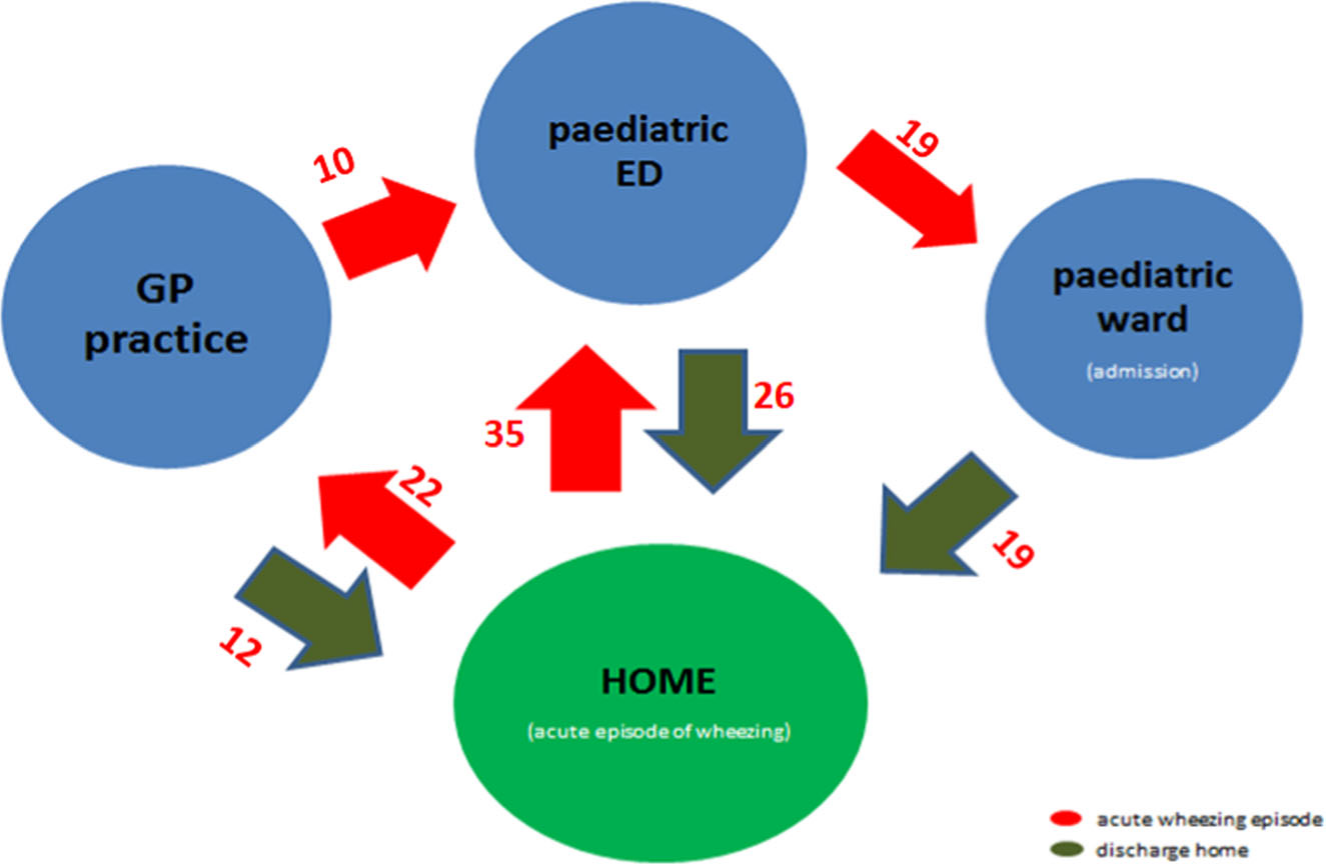
Process map describing patients’ journey during the first episode of wheeze they recall needing to seek for medical support—the red colour indicates admission with acute wheeze and the green colour indicates discharge—the number of parents who have described each journey are added next to each arrow

**Fig. 2:**
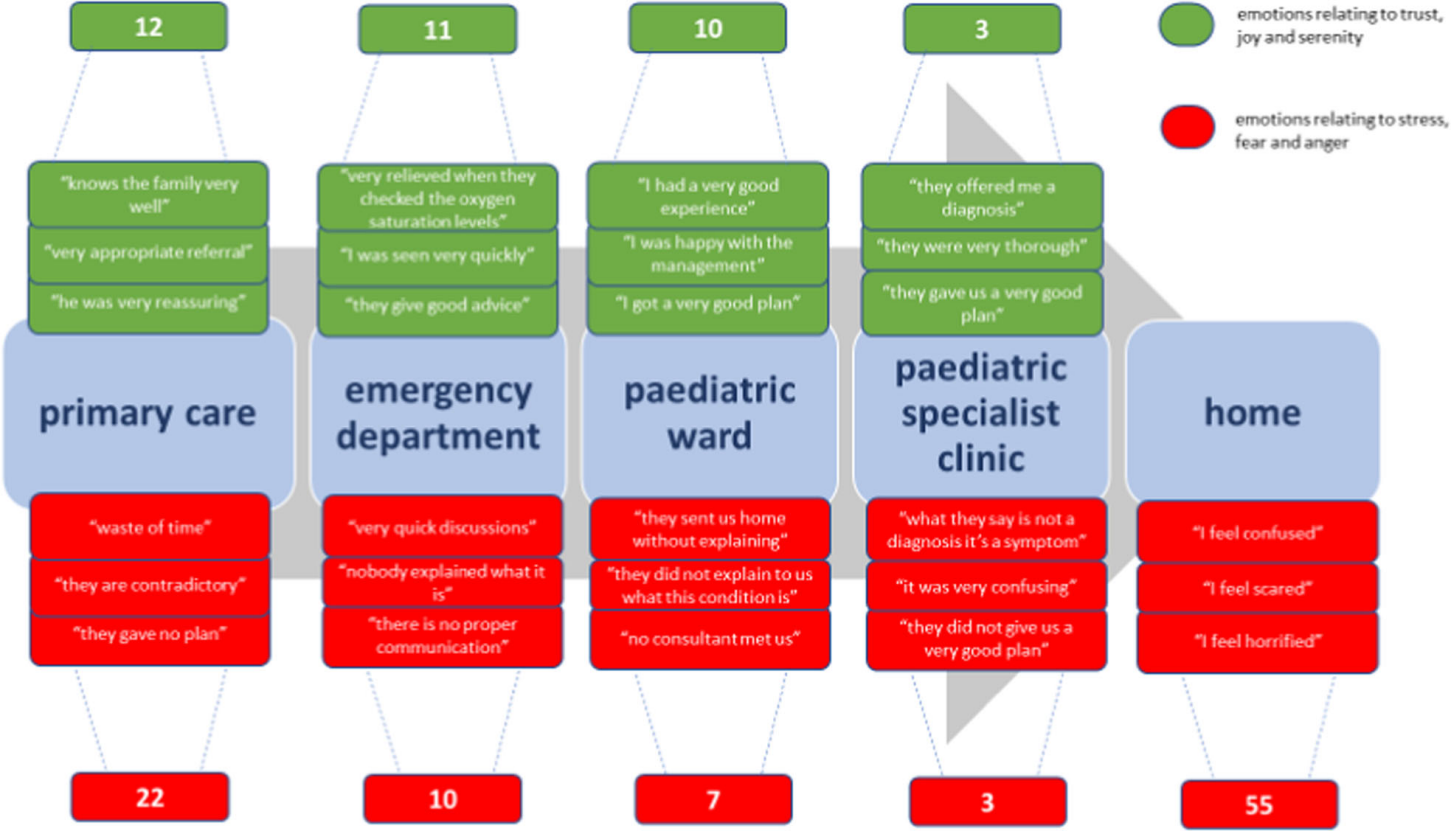
Emotional map describing parental feelings about healthcare services at each point of the journey of their child’s first episode of wheeze—number indicates frequency of parents expressing positive or negative emotions in each setting

The majority of parents were women (96%) with a mean age of 30.7 years (standard deviation (SD) 4.7). Based on parents’ narratives around their first experience of having a wheezing child, a process map was created to illustrate parents’ journey (Fig. 1)^16^ Parental emotions were mapped in a chart (Fig. 2) and seven main outcomes were identified through discussions. Notably, while most of the journey engendered a mix of emotions, both positive and negative, parents describe only negative emotions (stress, anger and fear) when they are at home and need to manage their wheezy child.

Personalised outcomes deriving from thematic analysis are:

a. Sense of reassurance

Parents value significantly having a sense of reassurance. This applies to both cognitive (knowing more) and affective (doctors feel for what they are going through) reassurance^17^.

Parent: “*more knowledge around the condition would provide reassurance*”

Parent: “*would feel less anxious if the team was more supportive*”

b. Less time off work

Parents feel that it is important to have less time off, and less impact on, work.

Parent: “*needs time free for work”*

Parent: “*I need to be able to concentrate at work”*

c. Family quality time

Improved family quality time is something that parents feel they miss.

Parent: *“I would like to have more relaxing days with the kids”*

Parent: *need “better family time”*

d. Parental mental health well-being

Parents feel they need to maintain their mental-health wellbeing.

Parent: “*I can’t stay calm and I feel always stressed*”

Parent: “*since he started having the wheeze I feel depressed*”

e. School readiness

Parents are concerned that they need to have some time to focus on their child’s development.

Parent: “*my niece is far more advanced than him*”

These outcomes are further described in Table 1 below.

**Table 1:** Main personalised outcomes presented as themes with accompanying sub-themes and quotes

Themes	Sub-themes	Quotes
Sense of reassurance	Cognitive reassurance	“I would feel less anxious”
		“I would feel less anxious and more reassured”
		“I’d feel more reassured in knowing there was a team I could contact”
		“I would also feel more confident in general decision making when he becomes unwell”
		“knowing more about it would provide reassurance”
		“learning more would make me feel more reassured”
	Affectionate reassurance	“I’d feel more reassured in knowing there was a team I could contact”
		“I would also feel more confident in general decision making when he becomes unwell”
		“I would feel less anxious if the team was supportive”
		“If I was more reassured I would be able to give the inhalers”
		“I need to feel more secure that he will be fine”
Sense of expertise	Education around the condition	“safety and knowledge are most important”
		“important is to able to give the inhalers”
		“safety and control of the situation are important”
		“it is important to know if his wheezing is life threatening”
		“I want to have more knowledge about the disease”
		“it would provide information on where to go and what to do”
		“this would give us more knowledge and would make us less stressed”
		“it is important to have knowledge about how serious it can be”
		“I need to have a better idea of what this is in order to feel better”
	Navigating healthcare services	“it would provide a feeling of knowing where to go and what to do”
		“it would provide information on where to go and what to do
Less time off work	Absenteeism	“I need to feel security, time free for work”
		“I want to have less days off work and more quality time with the family”
		“I need to get more days at work”
		“I need more time for work, I have no time for education, I need something simple to have at home”
	Effectiveness	“I am very stressed and not able to concentrate at work”
		“I need to be able to concentrate at work”
		“I need to get more meaningful days at work”
		“Important is to have knowledge and good planning and be more able to focus on other things like work”
Family quality time		“I want to get more time with the family”
		“I want to have a better life without stress”
		“I need not to catch viruses from the hospital”
		“I need to have more relaxing days with the kids”
		“I need to feel less stressed, have better family time”
		“To make life easier, I feel like if I’d have someone to talk to I would feel less worried”
		“I need to have less stress and a better quality of life”
		“I need to save time and effort and focus on family”
Coordination and continuity of care		“I need one single plan not ten”
		“I need a plan for his management that doesn’t change all the time”.
		“I need to have a better plan – to know whom to see”
		“I want to know that I have a concise plan”
		“I want to get what I need from all the doctors/not one here one there”
		“I want to see his doctors together”
		“I want to get one advice not ten”
		“I will have less time to lose if I see his doctors together”
		“I need to get one good advice not hundreds”
		“I need to know whom to follow the GP or the doctors?”
		“I need to feel more knowledgeable, to know which plan to follow”
		“I need to get a single opinion, to understand what this is”
		“I need to get a good doctor who sees him all the time”
		“I need not to go to different doctors all the time”
		“I need to get one final plan not different ones by different people”
Mental health well-being		“I need to maintain my mental health wellbeing—I can’t stay calm and I feel always stressed”
		“Since he started having the wheeze I feel depressed”
		“To feel less stressed and yes to have better mental health”
		“As R said to be less stressed, to have a better mental status, not to feel so stressed”
School readiness		“To be able to potty train him earlier—to get him ready to start reception”
		“I need to have some time to teach him things—my niece is far more advanced than him”

f. Sense of expertise

The need for an improved sense of expertise is highlighted by many parents.

Parent: *need for further “knowledge about the disease*”

Parent: *wanting to know how to navigate the healthcare services, knowing “where to go and what to do” when her child becomes wheezy*

g. More coordinated care

The most commonly suggested outcome points to the way healthcare services are designed and has to do with improved coordination of care.

Parent: *“I would like one single plan not ten”*

Parent: *“I would like a plan for his management that doesn’t change all the time”.*

## Discussion

This is the first study attempting to co-design personalised outcomes in preschool children with recurrent wheeze. These findings will inform the design of a tool that can be used in management of these patients and as another outcome in randomised controlled clinical trials.

A strength of this study is its use of an open survey tool to elicit parental feelings, unlike others who have used preselected criteria^14^ Although the study population was small, it is one of the largest described compared with similar studies^14^ Although we made every effort to recruit from varied settings, there should be caution in extrapolating the findings, in particular to tertiary care. Further work is required to design and validate an instrument based on these outcomes which could be subsequently tested in a larger population.

Our study highlighted coordination of care and communication between healthcare professionals as one of the main concerns of families. Suggested management plans often differed significantly between healthcare professionals, leading to inconsistent information being given to parents, potentially leading to poor continuity of care.

Parents highlighted the importance of health literacy, in particular how they can assess severity during an attack, and the navigation of healthcare services. It is important for parents to know when to escalate their child’s management and to choose the appropriate healthcare setting. Knowledge reduces stress and anxiety levels. It is especially challenging for immigrant parents to know where to turn for help when concerned enough to leave home. Although the increasing use of digital health aims to educate users, including non-native residents, around healthcare services^18^, many families remain confused. Also, parents value significantly having a sense of reassurance. This applies to both cognitive (knowing more) and affective (doctors feel for what they are going through) reassurance^17^.

Finally, quality family time and parents’ working schedules are severely affected by ill health in children. Measuring this aspect as a specific outcome will be helpful.

We have determined seven outcomes which may be important to parents of children with preschool wheeze. The next steps are to develop a formal score and test its sensitivity to intervention.

## Methods

Parents of preschool children with a history of at least one episode of recurrent wheeze were recruited in three different settings—emergency department, children’s ambulatory unit of Imperial College NHS Trust and general practices in the surrounding area that are part of Connecting Care for Children model (https://www.cc4c.imperial.nhs.uk/). The recruitment was either during opportunistic visits of the research team members to the emergency department and the children’s ambulatory unit or by general practitioner led identification, information and recruitment. Similar numbers of participants were interviewed at an emergency department (*n* = 24) and at a primary care or “supporting primary care” setting (*n* = 33). It is of note that 10 out of the 24 parents interviewed at an emergency department declared that their children are followed up in tertiary care clinics because of severe episodes of recurrent wheeze. Therefore parents of children with mild, moderate and severe forms of the condition are represented.

Parents, who consented to being contacted again, were invited to participate in two workshops held in a focus group format. The first explored (*n* = 11) further personalised outcomes, and the second (*n* = 9) refined and finally agreed the outcomes. All workshop participants had been interviewed by members of the team; eight out of the nine participants in the second workshop had participated in the first workshop. It is of note that both in semi-structured interviews and in focus group discussions parents were not given a list of questions describing their emotions, which we note was done in previous study published aiming to develop a relevant tool^14^ Feelings were not specifically named, but we aimed to highlight these by analysis of the in-depth discussions.

Main demographic data were analysed quantitatively. Each question was used to organise acquired data into categories (codes). Focus group discussions were not further segmented for analysis. All coding was carried out by two researchers independently (EK and HM) and coding consistency was compared and cross-checked for accuracy until a consensus was reached. Themes were identified from the coding. Inductive thematic saturation was reached. Themes were presented using continuous text with direct quotations to illustrate results. In addition, absolute frequencies and descriptive group comparisons were performed. NVivo 11.0 software was used for the building of the coding and the segmentation of units of coding but not for the presentation of the data.

### Reporting Summary

Further information on experimental design is available in the Nature Research Reporting Summary linked to this article.

## Supplementary information

Reporting Summary

## Data Availability

The authors declare that [the/all other] data supporting the findings of this study are available within the paper [and its supplementary information files].

## Supplementary information

**Supplementary Information** accompanies the paper on the *npj Primary Care Respiratory Medicine* website (10.1038/s41533-019-0120-3).
